# Descriptive analysis of preschool physical activity and sedentary behaviors – a cross sectional study of 3-year-olds nested in the SKOT cohort

**DOI:** 10.1186/s12889-017-4521-3

**Published:** 2017-06-30

**Authors:** Niels Christian Møller, Line B. Christensen, Christian Mølgaard, Katrine T. Ejlerskov, Karin A. Pfeiffer, Kim F. Michaelsen

**Affiliations:** 1 Department of Sport Science and Clinical Biomechanics, Research Unit for Exercise, Epidemiology and Centre of Research in Childhood Health, University of Southern Denmark, Campusvej, 55 Odense M, Denmark; 2 Department of Nutrition, Exercise and Sports, Faculty of Science, University of Copenhagen, 1958 Frederiksberg, Denmark; 3 Department of Kinesiology, Michigan State University, 308 West Circle Drive, 27R Intramural Rec Sports- Circle, East Lansing, MI 48824 USA

**Keywords:** Young children, Toddler, Recommendations, Objective monitoring, Accelerometry, Actigraph, Health behavior

## Abstract

**Background:**

Further collection of surveillance data is warranted, particularly in preschool populations, for optimizing future public health promotion strategies. This study aims to describe physical activity (PA) and sedentary behavior (SB) across different settings, including time in and out of daycare, and to determine the proportion of children complying with suggested PA recommendations in a high income country.

**Methods:**

Valid PA was assessed in 231 children (36.4 ± 1.1 months) with the Actigraph GT3X accelerometer, and information regarding date and time of dropping-off/picking-up children in daycare was provided by parents. Mean total PA (i.e., counts per minute (CPM)), moderate-to-vigorous physical activity (MVPA), SB time, and non-SB time was generated and compared across settings. Post hoc, PA and SB were examined in subgroups of low-active (1st quartile) and high-active (4th quartile) children.

**Results:**

Overall, boys and girls spent 1.4 ± 0.3 h/day and 1.2 ± 0.4 h/day in MVPA, respectively. Likewise, boys and girls accumulated 6.7 ± 0.8 h and 6.8 ± 0.9 h of SB time per day, respectively. Higher PA levels consistently co-occurred with lower SB time in the daycare setting. Girls accumulated less SB time in daycare than before and after daycare (β = −12.2%, *p* < 0.001 & β = −3.8%, *p* < 0.001, respectively). In boys, daycare-days contained more PA and less SB than non-daycare-days (CPM: β =29, *p* = 0.046, %MVPA: β = 0.83, *p* = 0.007, %SB: β = −2.3, *p* < 0.001, respectively). All children fulfilled recommendations of at least 3 h of daily non-SB. Eighty-nine percent of boys and 72% of girls met the daily 1-h MVPA recommendation for 5 year-olds. Lower proportions of children, especially boys, fulfilled MVPA recommendation on days with no daycare attendance. Generally, large mean differences in MVPA and SB were observed across all settings between the most active and the least active children, and only 7% of the low-active girls and 59% of the low-active boys fulfilled MVPA recommendations.

**Conclusions:**

Overall, the majority of children fulfilled MVPA guidelines for 5 year-olds, and all children complied with suggested recommendations of 180 min of daily activity. Daycare time was found to represent an important setting for PA. Substantial and consistent differences observed in the amount of time spent physically active between high- and low-active children across all settings indicate substantial variations in young children’s PA levels irrespective of the context.

**Electronic supplementary material:**

The online version of this article (doi:10.1186/s12889-017-4521-3) contains supplementary material, which is available to authorized users.

## Background

Overweight and obesity in children has increased over previous decades [1]. According to the World Health Organization, more than 41 million children under the age of 5 years are overweight [2]. Research indicates that higher physical activity (PA) during the preschool years (3–5 years) is associated with beneficial effects, not only with respect to adiposity and cardiometabolic indicators but also regarding motor skill development and social- and psychological health [3]. In accordance with this, sedentary behavior (SB) has been observed to be linked with less favorable health indicators, such as lower measures of psychosocial- and cognitive development in 0–4 year-olds [4].

PA has been reported to track through childhood and into adulthood [5, 6], thereby indicating the importance of early PA as an important health investment in childhood. However, the evidence of associations between PA and health is less extensive in preschoolers compared to school-aged children and adults, and researchers have called for more data linking PA to various health outcomes in order to optimize PA guidelines in preschoolers [7]. Thus, PA recommendations for children younger than five years have only been formulated fairly recently, and they are generally based on the pragmatic belief that the preschool years constitute a critical period for increasing PA and reducing SB that is pivotal for enhancing various health outcomes. Accordingly, there appears to be some consensus across the UK [8], Australia [9], and Canada [10] that preschoolers should accumulate at least 3 h of PA per day, with the latter further clarifying that children should progress towards at least 60 min of moderate-to-vigorous physical activity (MVPA) per day at the age of five years. Consequently, there are currently no quantifiable MVPA recommendations for children under the age of five years even though some have advocated that 1 h of structured PA together with 1 h of unstructured PA should be accumulated each day [11].

The focus on the importance of PA in preschoolers has increased during recent years, but information is lacking regarding how much PA and SB these young children are accumulating and in which settings such accretion primarily takes place. Since the vast majority of preschool-aged children spend considerable time in institutions in the form of kindergartens or daycare centers, the out-of-home preschool environment seems to be an ideal setting for PA promotion. Interest in institutional setting as a potential important early-life correlate of PA, therefore, has logically emerged [12]. Accordingly, potential correlates of SB have typically been examined separately in the home physical and preschool/childcare center environments, but largely inconclusive results have been produced [13].

The collection of surveillance data is an important part of public health promotion strategies, and further monitoring of PA during early life may serve to identify potential issues that could help optimize public health initiatives. Detailed knowledge in terms of when and where toddlers and preschoolers are sedentary and physically active, including institutional time and leisure time, is pivotal for the initiation, evaluation, and adjustment of future programs launched to increase PA in young children. Therefore, the purpose of this study was to provide an overview of levels and distributions of PA and SB among healthy 3-year-olds in a high income country. This was done by: 1) describing the distribution of total PA, SB time, non-SB time, and MVPA time across typical everyday settings, 2) applying suggested recommendations to determine the proportion of children meeting current PA guidelines. Furthermore, we estimated the proportion of children accumulating at least 60 min of daily MVPA.

## Methods

### Recruitment and participants

Nested cross-sectional data used in this study are from the 3-year examination in the longitudinal observational SKOT cohort study, which has been described in detail previously [14, 15]. In short, based on random selection from the National Danish Civil Registry of infants born in the greater Copenhagen area, 330 children were enrolled in the study between April 2007 and May 2008 at age 8.5 months and examined at 9, 18, and 36 months. Inclusion criteria were being a singleton infant born at term (≥37 weeks of gestation), with no diseases expected to affect growth or nutritional intake. Anthropometric measures and questionnaire data were collected at all examinations. Twenty children were lost to follow-up before age 3 years, and one child was excluded due to severe chronic disorder with late manifestation. The remaining 309 children still eligible for participation were invited to the 3-year examination. All 3-year examinations (± 3 months) took place at the Department of Nutrition, Exercise and Sports, Frederiksberg, Denmark during the period from October 2009 to October 2010. Two-point-6 % of the children attended daycare in private homes, 12.6% attended day nursery, and 84.8% attended kindergarten (preschool) – together they are referred to as children attending daycare.

### Anthropometrics

Body weight and height were measured according to standard procedures, as described elsewhere [14, 15]. Means of available measures were used in all analyses. Age- and sex-specific Z-scores for body mass index (BMI-Z) were calculated by the software WHO Anthro (Department of Nutrition, World Health Organization, Geneva, Switzerland). The proportions of children who were overweight or obese were calculated according to sex- and age specific body mass index (BMI) cut-points [16].

### Socio-economic status, sleep and daycare attendance

A proxy of socio-economic status was obtained through information on mothers’ level of education in a parent-completed questionnaire. We defined educational categories based on length of completed academic education, completion of vocational education or training, and gymnasium/high school or lower secondary school education. In this Danish context, academic education refers to higher educations at universities or university colleges, vocational education or training refers to education offered at special state-funded vocational schools, gymnasium/high school refers to a 3-year academic-oriented upper secondary educational programme, and lower secondary school refers to education provided in state schools or private schools from Year 0 to Year 9 or 10. Information was also gathered regarding night sleep (“At what time does your child usually fall asleep in the evening?”, “At what time does your child usually wake up in the morning?”), and day naps (“How many days per week does the child sleep during the day?”, “For how long does the child sleep on days where the child sleeps during the day?”).

Information regarding time and date of dropping-off/picking-up children from daycare and date of any sick days was obtained from a parent-completed log.

### Physical activity

#### Instrument

Physical activity was measured with the ActiGraph GT3X accelerometer (Pensacola, FL, USA). The research staff personally fitted all children with the accelerometer. Parents and children were instructed on how to wear the accelerometer (on the right hip using an elastic belt for at least 7 days and nights) and were told to remove it only during water-based activities (bathing, swimming, etc.). The accelerometers were returned to the researchers in a prepaid envelope.

#### Processing of physical activity data

The processing of PA data was conducted using the software packages Propero version 1.1.1 (Department of Sport Science and Clinical Biomechanics, Research Unit for Exercise Epidemiology and Centre of Research in Childhood Health, University of Southern Denmark) and Actilife version 6.4.5 (Pensacola, FL, USA).

Counts per minute (CPM) were used as an estimate of mean total PA, and cut-offs for SB (which simultaneously define time spent in any activity of at least light intensity - i.e. non-SB time), and MVPA were <25 counts/15 s [17] and ≥420 counts/15 s [18], respectively. The accelerometer data were sampled in 2-s epochs and re-integrated into 10-s epochs, and cut-points were scaled accordingly by applying a conversion factor of 0.67. Outcome variables for total valid wear-time, mean total PA, and time spent below or above each activity threshold, as appropriate, were generated by Propero software for each typical everyday setting. The proportions of SB time, non-SB time, and MVPA time are presented both as a percentage of total wear time and as absolute hours/day to eliminate the influence of total wear time and to ease interpretation and comparisons with other studies.

Accelerometer non-wear was defined as periods of 20 min or more of consecutive zeroes. These periods were removed before data analysis. The minimum requirement for wear time inclusion was 4 valid monitoring days, each valid day containing at least 8 h of valid PA assessments.

### Differentiating physical activity during waking hours from body movements during sleep

In this study, continuous 24-h accelerometer data were recorded, resulting in a need to separate PA during waking hours from sporadic movements made during night-sleeping. We used Individual Manual Inspection (IMI) to identify specific times of waking up in the morning and falling asleep in the evenings for each child for each day of monitoring. This method involved manual visual inspection of activity graphs produced by the sleep analysis module in the Actilife software. Falling asleep was defined as the time during which more continuous patterns of PA were followed by at least 5 min of zero counts and an obvious change in behavior pattern to a few single volatile sporadic movements, thereby allowing for tossing and turning in bed. Likewise, wakeup time was defined as the time where more steady patterns of PA followed at least 5 min of zero counts, with allowance given for single sporadic movements during sleep prior to the 5 min zero counts. The visual inspection was performed twice by two trained research assistants and the results were compared. Scoring differences of more than 10 min were double-checked and reevaluated by the second research assistant and LBC. Only PA occurring during waking-hours, as identified by morning wake-up and times of falling asleep at night, was included in the analyses. We used information obtained in the parent-completed questionnaire to compare morning wake-up time and sleep time in evenings as identified by the IMI and parents’ reports, respectively. Additionally, we used the filtering of 20 min or more of consecutive zero counts to exclude sleep time in children who were napping during the daytime.

### Defined everyday settings

Based on accelerometer data defined by the use of the Individual Manual Inspection approach and information on daycare attendance provided by parents’ logs, time-stamped data were analyzed separately for predefined typical settings of the day and week (i.e., all days overall, sick days, daycare-days (DC-days), time before daycare, time in daycare, time after daycare, and non-day-care-days (non-DC-days). All children except one were enrolled in childcare, but some of the children reported having some weekdays without daycare attendance, and 40 children did not report any days in daycare during the monitoring period. These children may have worn the accelerometer during days off or during non-typical weeks like vacation time. Weekdays where parents reported their child did not attend daycare had more similarities with weekend days than with weekdays where parents reported that children where in daycare (data not shown). Accordingly, days were recoded as either DC-days or non-DC-days.

### Data analyses and statistics

All accelerometer outcomes were processed separately across the following domains: total time all days, sick days, DC-days, time before daycare, time in daycare, time after daycare, and non-DC-days. Crude hourly mean total PA intensity was processed to illustrate more general PA levels throughout the day. To assess if PA or SB differed across settings – (e.g., when children were in daycare and not in daycare), linear regressions were performed with the relevant accelerometer output included as the dependent variable. “Settings” were treated as a dummy variable and included as independent variable in the model. “Cluster option” in Stata IC 14.0 was used on child ID to obtain robust standard errors thereby accounting for repeated observations. The statistical significance of the overall influence of the different settings was evaluated using a Wald test. In case of overall significant influence due to settings, we post hoc used the “lincom” command in Stata to examine if PA or SB outcomes differed across the following combinations of settings: 1) time before daycare vs. time in daycare, 2) time before daycare vs. time after daycare, 3) time in daycare vs. time after daycare. Furthermore, potential differences were examined between DC-days and non-DC-days.

Differences in proportions of children meeting PA recommendations across settings and sex were examined based on the chi-square test. Quartiles based on mean total PA (CPM) were generated post hoc, and subgroups of low active (1st quartile of CPM) and high active (4th quartile of CPM) children were compared for MVPA and SB. Analyses were restricted to individuals with complete data and statistical significance was based on α = 0.05.

## Results

### Description of the sample

Participant characteristics are presented in Table 1. Two-hundred-sixty-four children completed the 3-year examination, whereas 7 withdrew from the study before the 3-year examination, 12 children did not respond to the invitation, and 26 children were unable to participate. Four accelerometers were lost in the mail or not returned by the families, and 29 children provided less than 4 valid days of monitoring. No instrument malfunction occurred.

**Table 1:** Participant characteristics

	Total (*n* = 231)	Girls (*n* = 117)	Boys (*n* = 114)	*P*
Age (months)	36.4 (1.1)	36.3 (1.0)	36.5 (1.2)	0.3
Weight (kg)	14.5 (1.5)	14.2 (1.5)	14.8 (1.5)	0.002
Height (cm)	95.7 (3.4)	94.8 (3.2)	96.6 (3.4)	0.0001
BMI (kg*m^−2^)	15.8 (1.1)	15.8 (1.2)	15.9 (1.1)	0.6
BMI-for-age-Z-scores	0.23 (0.9)	0.25 (0.9)	0.20 (0.83)	0.7
Overweight/obese (%)	6.5/0	9.4/0	3.5/0	0.07/na

*BMI* Body mass index. Data are means and SD unless otherwise explained

Of the children’s mothers, 40.7% had long academic education (>4 years), 35.5% had medium length academic education (3–4 years), 11.3% had short academic education (<3 years), 8.7% had vocational education or training, and 3.9% had no education above gymnasium/high school or lower secondary school. The 231 children (87.5%) who provided valid PA data were slightly lighter (14.5 ± 1.5 kg vs. 15.0 ± 1.5 kg, *p* = 0.0018), shorter (95.7 ± 3.4 cm vs. 97 ± 3.5 cm, *p* = 0.04), and had a lower BMI (15.8 ± 1.14 vs. 16.5 ± 0.96, *p* = 0.006) compared to the children who did not provide valid PA data, but they did not differ significantly by age, sex, overweight/obesity status, or maternal educational level (Additional file 1: Table S1).

Overall, children had a mean of 7.0 ± 1.1 valid monitoring days and on average 12.6 ± 0.8 h of valid wear time per day. On average, children attended daycare 4.1 ± 1.2 days, and daily valid wear time while in daycare was 6.4 ± 1.2 h (Table 2). Children were dropped off in daycare at 8:32 AM ±42 min and picked up at 3:31 PM ± 43 min, and thus spent an average of 6.98 ± 1.08 h per day in daycare. There were no significant differences in valid wear time by sex, reporting of any days in daycare, or napping during the day (Additional file 2: Table S2).

**Table 2:** Wear-time across settings by type of days and monitoring week

	*n*	Hours, total	Days	Hours/day
All days, overall	231	88.2 (15.0)	7.0 (1.1)	12.6 (0.8)
Sick days	21	18.1 (9.4)	1.5 (0.8)	12.3 (1.7)
Monitored during regular weeks				
All days, overall	191	87.9 (14.9)	7.0 (1.1)	12.6 (0.8)
DC-days	191	51.1 (16.0)	4.1 (1.2)	12.6 (1.0)
DC-days - before DC	189	6.8 (3.4)	4.1 (1.2)	1.6 (0.6)
DC-days - in institution	191	26.4 (9.5)	4.1 (1.3)	6.4 (1.2)
DC-days - after DC	190	18.0 (6.4)	4.0 (1.2)	4.5 (1.0)
Non-DC-days	185	36.2 (15.8)	2.9 (1.2)	12.6 (1.0)
Monitored during irregular weeks (no days in DC)				
All days, overall	40	89.4 (14.8)	7.2 (1.1)	12.5 (0.9)

*DC* daycare. Data are means and SD

Overall, boys and girls spent an average of 1.4 ± 0.3 h/day (11.2 ± 2.6% of daily time) and 1.2 ± 0.4 h/day (9.7 ± 2.9% of daily time) in MVPA, respectively. Likewise, boys and girls accumulated 6.7 ± 0.8 h (53.5 ± 5.1% of daily time) and 6.8 ± 0.9 h (54.7 ± 5.9% of daily time) of daily SB time, whereas 5.9 ± 0.7 h (46.5 ± 5.1% of daily time) and 5.7 ± 0.8 h (45.3 ± 5.9% of daily time) of non-SB time were accumulated in boys and girls (Table 3).

**Table 3:** Physical activity and sedentary behavior across settings

		Mean total PA	Sedentary behavior		Non-sedentary behavior		MPVA	
	*n*	CPM	Hours/day	%	Hours/day	%	Hours/day	%
Boys^╫^								
All days, overall	114	584 ± 124^*^	6.7 ± 0.8	53.5 ± 5.1	5.9 ± 0.7	46.5 ± 5.1	1.4 ± 0.3	11.2 ± 2.6^*^
Sick days	10	442 ± 180	7.4 ± 1.3	60.6 ± 10.5	4.9 ± 1.5	39.4 ± 10.5	1.1 ± 0.6	8.5 ± 4.5
DC-days	95	593 ± 131_c_^*^	6.6 ± 0.8	52.4 ± 5.4_c_	6.0 ± 0.8	47.6 ± 5.4_c_	1.4 ± 0.4	11.6 ± 2.9_c_^*^
Before DC^**^	94	376 ± 20	1.0 ± 0.4	63.3 ± 7.0	0.6 ± 0.3	36.7 ± 7.0	0.1 ± 0.06	6.8 ± 2.8^*^
In DC	95	654 ± 175_a_^*^	3.1 ± 0.6	47.9 ± 7.9_a_^*^	3.4 ± 0.9	52.1 ± 7.9_a_^*^	0.9 ± 0.3	13.0 ± 4.2_a_^*^
After DC	95	572 ± 223	2.4 ± 0.6	55.5 ± 6.7	2.0 ± 0.5	44.5 ± 6.7	0.5 ± 0.2	11.0 ± 3.4
Non-DC-days	112	564 ± 147	6.9 ± 1.0	54.7 ± 6.1	5.7 ± 1.0	45.3 ± 6.1	1.4 ± 0.4	10.8 ± 3.0^*^
Girls^╫^								
All days, overall	117	527 ± 129	6.8 ± 0.9	54.7 ± 5.9	5.7 ± 0.8	45.3 ± 5.9	1.2 ± 0.4	9.7 ± 2.9
Sick days	11	318 ± 121	8.1 ± 2.2	65.2 ± 9.9	4.2 ± 1.2	34.8 ± 9.9	0.6 ± 0.3	5.3 ± 2.5
DC-days	95	543 ± 144	6.8 ± 1.1	53.8 ± 6.8_c_	5.8 ± 1.0	46.2 ± 6.8_c_	1.3 ± 0.4	10.1 ± 3.1_c_
Before DC^**^	95	351 ± 116	1.0 ± 0.4	63.4 ± 8.5	0.6 ± 0.3	36.6 ± 8.5	0.1 ± 0.06	6.0 ± 2.5
In DC	96	580 ± 201_b,d_	3.2 ± 0.7	51.2 ± 9.3_a_	3.1 ± 0.9	48.2 ± 9.3_a_	0.7 ± 0.3	10.8 ± 4.3_b_
After DC	95	541 ± 169	2.5 ± 0.8	55.0 ± 8.1	2.1 ± 0.6	45.0 ± 8.1	0.5 ± 0.2	10.2 ± 3.7
Non-DC-days	113	523 ± 148	7.0 ± 0.9	55.4 ± 6.0	5.6 ± 0.9	44.6 ± 6.0	1.2 ± 0.4	9.5 ± 3.1

*CPM* counts per minute, *MVPA* moderate-to-vigorous physical activity, *DC* daycare

^╫^: significant influence of settings on levels of all outcomes examined, ^*^: significant sex differences, ^**^: least active setting with the exception of sick days, _a_: significant different from after DC and before DC, _b_: significant different from before DC, _c_: significant different from non-DC-days, _d_: borderline significant different from after DC. ^**╫**)^, ^**)^, _b)_: all *p*-values < 0.001, ^*)^: all *p*-values < 0.03, _a)_: all *p*-values < 0.006), _c)_: all *p*-values < 0.05), _d)_: *p* = 0.08

There were no differences between children’s wake up time according to the IMI approach and parents’ reports, respectively (06:47:31 AM ±31.7 min vs. 06:49:06 AM ±40.1 min, *p* = 0.55). However, children fell asleep later based on the IMI approach compared to parent provided information (asleep: 20:35:28 PM ± 46.6 vs.19:58:31 PM ± 32.8 min, *p* < 0.0001).

### Hour-by-hour total PA

**Fig. 1:**
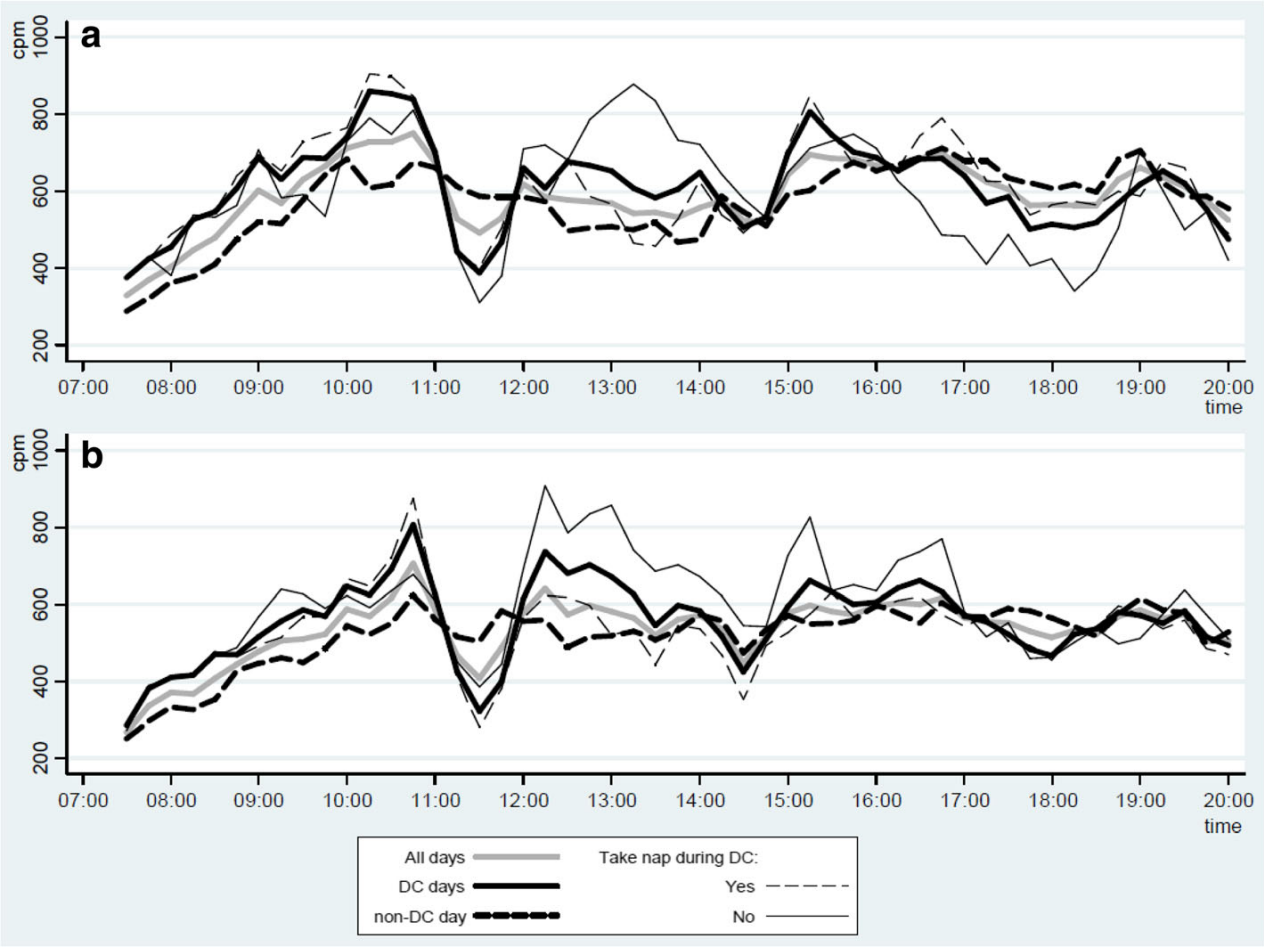
Mean total physical activity (CPM) during waking hours for (**a**) boys and (**b**) girls. Only hours were at least 80% of the study sample was awake and recorded valid data are shown. CPM: counts per minute, DC: Daycare

Crude hourly mean total PA level (CPM) revealed patterns of low PA levels around 11:30 AM, 02:30 PM, and 06:00 PM on DC-days. Furthermore, on DC-days, differences in PA patterns were observed between boys taking a nap and boys not taking a nap during early (12:30 PM-02:00 PM) and late (04:00 PM-07:00 PM) afternoon. In girls, this phenomenon could only be observed during the early afternoon (12:00 PM-02:30 PM) (Fig. 1). Post hoc sub-analyses supported these findings; overall, on DC-days, on non-DC-days, and in daycare time, the girls who took a nap registered lower mean total PA compared to those girls not taking a nap. Boys taking a nap were more physically active after daycare on DC-days compared to boys who did not take a nap (Additional file 2: Table S2).

### The effect of daycare time on children’s PA and SB

Significant differences among settings were observed for all PA and SB variables in both girls and boys (all *p* < 0.0001). Generally, post hoc analyses revealed that the daycare setting contributed to higher PA levels and lower SB than non-daycare. In girls, higher total PA (CPM) was obtained during daycare compared to time before and after daycare (β = 228, *p* < 0.001 & β = 39, *p* = 0.08, respectively). Furthermore, girls accumulated more time in MVPA in daycare compared to time before daycare (β = 4.8%, *p* < 0.001). Similarly, compared to time before and after daycare, lower amounts of SB time were accumulated during daycare (β = −12.2%, *p* < 0.001 & β = −3.8%, *p* < 0.001, respectively). Opposite results were observed for non-SB time. On days during which they attended daycare, girls engaged in significantly less SB but more MVPA compared to days without daycare attendance (β = −1.6%, *p* = 0.002 & β = 0.57%, *p* = 0.015). Compared to all other settings with the exception of sick days, girls were least active and accumulated most SB time in the morning before arriving to daycare.

Boys engaged in the highest level of total PA in daycare compared to time before and after daycare (β = 277, *p* < 0.001 & β = 82, *p* = 0.006, respectively). Furthermore, boys accumulated more time in MVPA during time in daycare compared to time before and after daycare (β = 6.2%, *p* < 0.001 & β = 2.0%, *p* < 0.001, respectively). Also, during time in daycare boys accumulated less SB time compared with time before and after daycare (β = −15.4%, *p* < 0.001 & β = −7.6%, *p* < 0.001, respectively). DC-days in boys consisted of more time spent in PA and less SB than non-DC-days (CPM: β =29, *p* = 0.046, %MVPA: β = 0.83, *p* = 0.007, %SED: β = −2.3, *p* < 0.001, respectively). As observed in girls, boys were least active and accumulated most SB time in the morning hours before arriving to daycare – this was true for all variables and settings with the exception of sick days.

Boys exhibited higher levels of total PA (CPM) on all days overall (*p* < 0,001), on DC-days (*p* = 0.01), and when in daycare (*p* = 0.007) when compared to girls. In daycare, boys also accumulated less SB time and more non-SB time compared to girls (*p* = 0.009). Furthermore, significant sex differences in MVPA were observed across all settings with the exception of sick days and time after daycare (all *p*-values < 0.03) (Table 3).

### Meeting PA recommendations and associations with daycare attendance

Overall, all boys and girls fulfilled the recommendation of at least 3 h of activity per day. For sick days specifically, these numbers decreased to 82% for girls and 90% for boys, respectively (Table 4). Overall, 89% of the boys met the recommendation for 5-year-olds of at least 1 daily hour of MVPA. A significantly (*p* = 0.001) smaller proportion (72%) of girls met this recommendation (Table 4). Furthermore, fewer girls than boys (*p* = 0.015) met the MVPA recommendations on DC-days. Generally, more children met MVPA recommendations on DC-days than on non-DC-days although these differences only were significant in boys (boys: *p* = 0.02, girls: *p* = 0.24). Finally, as expected, the lowest proportions of girls (18%) and boys (40%) were observed to fulfill MVPA recommendations on sick days (Table 4).

**Table 4:** Percentages of children meeting physical activity recommendations by type of days

	*n*	≥180 min non-sedentary per day	≥60 min MPVA per day
Total			
All days, overall	231	100	81
Sick days	21	86	29
DC-days	190	100	82
Non-DC-days	225	100	72^**^
Boys			
All days, overall	114	100	89^*^
Sick days	10	90	40
DC-days	95	100	88^*^
Non-DC-days	112	100	76^**^
Girls			
All days, overall	117	100	72
Sick days	11	82	18
DC-days	95	100	75
Non-DC-days	113	100	67

*MVPA* moderate-to-vigorous physical activity, *DC* daycare

^*^: significant sex differences, ^**^: significant differences between proportion of children meeting the recommendations on DC-days and non-DC-days. ^*)^, ^**)^: all *p*-values < 0.02

### PA levels and SB in low- and high -active children

Large differences in MVPA and SB were consistently observed across all settings between the most (4th PA quartile) and the least active (1st PA quartile) children. In contrast to the total sample significant sex differences were also observed in time after daycare (*p* = 0.04) and on non-DC-days (*p* = 0.0006) in the least active children. No significant sex differences were observed in any setting in the most active quartiles of boys and girls (all *P* > 0.11) (Table 5).

**Table 5:** Physical activity levels across settings in high- vs. low-active children

	Mean total PA (CPM)		Sedentary behavior (% of time)		MPVA (% of time)	
	Q1	Q4^**^	Q1	Q4^**^	Q1	Q4^**^
Boys^╫^						
All days, overall	441 ± 63^*^	742 ± 95	58.1 ± 4.1^*^	49.7 ± 5.1	8.1 ± 1.5^*^	13.8 ± 2.0
DC-day	447 ± 82^*^	754 ± 110	57.0 ± 4.1^*^	48.3 ± 5.0_d_	8.2 ± 2.2^*^	14.5 ± 2.5
Before DC^***^	306 ± 77	447 ± 141	66.5 ± 5.5	61.2 ± 7.3	5.3 ± 1.8	8.3 ± 3.5
In DC	488 ± 128^*^_a_	823 ± 178_a_	53.4 ± 8.1^*^_a,b_	43.3 ± 6.8_a,c_	8.9 ± 3.1^*^_a_	16.5 ± 4.2_a,e_
After DC	446 ± 104	750 ± 175	58.1 ± 5.7^*^	51.1 ± 8.5	8.4 ± 2.7^*^	13.8 ± 3.9
Non-DC-day	431 ± 73^*^	725 ± 151	58.7 ± 4.9^*^	50.7 ± 6.5	7.8 ± 1.7^*^	13.3 ± 2.7
Girls^╫^						
All days, overall	371 ± 50	693 ± 86	61.8 ± 3.6	49.1 ± 3.9	6.3 ± 1.3	13.2 ± 2.3
DC-day	379 ± 71	701 ± 113	61.5 ± 5.0	48.3 ± 4.5	6.6 ± 1.7	13.4 ± 2.4
Before DC^***^	296 ± 121	409 ± 98	67.4 ± 7.7	59.1 ± 7.2	5.0 ± 2.8	7.3 ± 2.4
In DC	384 ± 97_a_	773 ± 202_a_	60.0 ± 7.6_a_	45.4 ± 4.4_a,e_	6.6 ± 2.3_a_	14.7 ± 4.0_a_
After DC	390 ± 109	675 ± 183	62.1 ± 7.3	49.8 ± 7.6	6.9 ± 2.4	13.3 ± 3.7
Non-DC-day	369 ± 57	695 ± 130	61.6 ± 4.3	50.0 ± 5.1	6.3 ± 1.4	13.2 ± 2.9

*CPM* counts per minute, *Q1* 1st quartile (least active children), *Q4* 4th quartile (most active children), *MVPA* moderate-to-vigorous physical activity, *DC* daycare

^╫^: significant influence of settings on levels of all outcomes examined ^*^: significant sex differences. ^**^: significant differences between Q4 and Q1across all settings. ^***^: least active setting, _a_: significant different from before daycare, _b_: borderline significant different from after daycare, _c_: significant different from after daycare, _d_: borderline significant from non-DC-days, _e_: borderline significant from after daycare. ^*)^: all *p*-values < 0.04, ^**)^: all *p*-values < 0.003, _a)_: all *p*-values < 0.02), _b), d)_: *p* = 0.06, _c)_: *p* = 0.003, _e)_: *p* = 0.07

Overall significant influences of settings on MVPA and SB were observed for the most and least active girls and boys (all *p* < 0.03). Post hoc analyses revealed that both girls and boys in the fourth PA quartile accumulated significantly less SB time in daycare compared to time before daycare (girls: β = −13.6%, *p* < 0.001; boys: β = −17.9%, *p* < 0.001) and time after daycare (girls: β = −4.36%, *p* = 0.07; boys: β = −7.8%, *p* = 0.003). Compared to all other settings, both high- and low-active girls and boys participated in the least MVPA and most SB time during morning hours before daycare (Table 5).

Overall, only 7% of the least active girls and 59% of the least active boys fulfilled the recommendation for 5-year-olds of at least 1 h of MVPA per day. In high active girls and boys these numbers were 97% and 100%, respectively (data not shown).

## Discussion

Results showed clear differences in PA levels and SB across everyday settings, with children consistently being more active when in daycare. These findings illustrate the importance of investigating where and how children optimally should be active and how and where PA promotion programs for preschoolers best could be launched. Low levels of total PA and MVPA consistently co-occurred with high levels of SB in the morning before daycare attendance. Likewise, children were more active and accumulated less SB time on DC-days compared to non-DC-days, particularly during time spent in daycare. Similar patterns were generally observed across settings in high- and low-active children as observed in the total sample, although fewer significant differences were observed in these subgroups indicating more homogenous behaviors across settings in high- and low-active subjects.

### Daycare time and children’s PA and SB

We observed clear patterns of low PA on DC-days around 11:30 AM, 2:30 PM and 6:00 PM, possibly reflecting consistent meal times across DC-days and the daycare institutions. The forenoon (10:00 AM-11:00 AM) and early afternoon (12:00 PM-02:00 PM) were characterized by periods of high PA levels. This corresponds well with commonly scheduled playtime in Danish preschools. Although day-time-specific routines to some extent are expected to affect children’s PA level across different daycare centers (e.g., around lunch time), it is noteworthy how different daycares seem to follow the same hourly routines throughout the day. Such patterns did not appear on non-DC-days, indicating more flexibility across individual families on those days.

The fact that results revealed higher PA levels in daycare than out of daycare supports previous findings in preschoolers observed in a Danish study [19]. Olesen et al. reported lower PA levels in children during leisure time than during their preschool day. Furthermore, the authors noted substantially lower total PA levels on weekend days compared to weekdays. Interestingly, these differences were reported to change throughout the day (i.e., children were more active on weekdays compared to weekend days from 8 AM to 4 PM but more active in weekends from 4 PM to 8 PM). These findings correspond well to our findings of children being more active on weekdays during daycare hours. Generally, previous studies from other countries have reported some similar and some contradictory results when examining preschool vs. non-preschool PA by comparing weekdays and weekends [20–22].

High PA levels in the daycare setting are in contrast to findings observed in other countries when describing PA levels within the childcare setting [12]. Unlike our study, these observations, though, did not take into account the potential individual differential impact of time and place on children’s PA, although the authors noted that other data available suggest that the problem of low PA is not unique to the child care setting. Furthermore, these differences are in part likely due to heterogeneity between samples, differences in monitor type, measurement protocol (e.g., minimum criteria for valid day and time of start/stop in morning/evening). Hesketh et al. previously examined preschoolers’ PA in the UK by specifically exploring the potential differential impact of time and place including childcare. In accordance with our observations, they reported that young children accumulated more MVPA in childcare compared to when at home [23].

According to Danish daycare law, it is compulsory for all kindergartens and day nurseries in Denmark to create a written pedagogical curriculum plan describing targets for working with children within specific themes, including social competences and relations, nature, and the body and body movements. Typically, within the Danish context it is also mandatory for preschool children to spend time outdoors on a daily basis and outdoor environments, such as playground and portable equipment, are usually available. A recent review conducted by Tonge et al. identified size, use, and presence of outdoor environment as consistent PA and SB correlates in preschoolers [24]. Furthermore, outdoor childcare hours have been suggested to comprise more PA than indoor hours [25]. Other factors, such as presence of peers and peer prompts, in the daycare setting may contribute to our findings of elevated PA levels during daycare hours [24].

Maternal and paternal behaviors have previously been observed to associate with children’s home- and neighborhood-based SB and MVPA [26], and mothers’ SB during the morning period has related negatively to children’s MVPA [27]. We speculate that early morning time periods, especially during less flexible weekdays, are characterized by essential daily living activities (e.g., bathing, dressing, and eating) with little options for parents and children to be active before daycare attendance. Although the present study was not designed to identify specific PA- and SB-correlates but more should be seen as a descriptive hypothesis-generating study, our findings indicate that morning and afternoon during weekdays are obvious intervention opportunities in preschoolers. Promotion of PA and reduction of SB during early morning-hours seems difficult because of a need to perform practical tasks which might leave fewer opportunities to be physically active. Generally, there may be more potential for promoting parents’ and children’s home-based PA on weekdays in the afternoon after daycare or on weekends. It is possible that factors such as pedagogical curriculum and institutional policy, outdoor environment, and the potential importance of peers might influence attempts to increase childcare PA levels.

It is important to note that we observed considerable differences between low- and high-active children across all settings and PA variables examined. This is interesting and indicates that low-active children are substantially and consistently less active compared to highly active children, irrespective of the context. Most studies find that boys are more active than girls [28]; however, sex-based differences were not observed among the most active children in the current study. These findings support that targeting of PA and non-SB seems conceivable and should be favored across all settings in the least active children.

### Adhering to PA recommendations

Previous PA reviews conducted in preschoolers generally have confirmed low PA levels, high levels of SB [12], and low prevalence of children adhering to recommendations [29], or the authors have been unable to make conclusions regarding the PA levels due to methodological inconsistencies [30]. Cardon et al. [20] previously reported that only 7% of Dutch preschoolers engaged in 60 min of MPVA per day, but they used a cutoff point of ≤615 counts/15 s to define MVPA as suggested by Sirard et al. [31]. In our study, we reported that 72% of girls and 89% of boys accumulated at least 60 min of daily MVPA which is recommended for children when they reach the age of 5 years. However, when we tried to apply the Sirard cut-points [31] results became quite comparable to the findings observed by Cardon et al., as proportion of children complying with MVPA guidelines decreased substantially to 7% and 12%, respectively (results not shown). Similarly, we found that all children met the recommendation of 3 h of daily engagement in PA with any activity. However, when applying high SB cut-points, as suggested by Sirard et al. [31], only 1% of children in the present study fulfilled recommendations of at least 3 h PA with any intensity per day (results not shown). This illustrates the impact that the selection of cut-points will have on overall conclusions. We would argue that a fairly “low” cut-point to define SB seems preferable in young children. A good example of why this might be preferable is a case such as sitting on the floor instead of on a chair, which often involves small movements to shift position. These small movements may be regarded as small breaks in SB time. Considering the intermittent nature of young children’s PA [32], free play activities in young children could be hypothesized to involve a high frequency of such breaks even though the overall activity (lying or sitting) in itself may qualify as SB according to some observational protocols (e.g., the Children’s Activity Rating Scale, CARS [33]). The use of ≤25 counts/15 s and ≤420 counts/15 s cut-points to define SB and MVPA, respectively, as applied in the present study, has previously been suggested by Trost et al. as the best cut-off thresholds to use in young children [34].

In the present study, both boys and girls were observed to accumulate most of their time in MPVA when in daycare (boys, 13% of daycare time and girls 10.8%). These numbers correspond well to previous results observed in US preschoolers indicating that 3–5-year-olds engage in 7.7 min of MVPA per hour of preschool attendance, corresponding to approximately 13% of the time [35].

We found that only 7% of the least active girls and 59% of the least active boys fulfilled the recommendation of at least 1 h of MVPA per day. Although MPVA recommendations do not apply for children younger than 5 years, these are discouraging results from a public health perspective. This, especially since early PA levels during childhood have been reported to be predictive for later PA levels [5, 6], and since risk factors for lifestyle-related diseases tend to cluster in the least fit and least active children [36]. Therefore, the least active children even in daycare or kindergarten are most likely in most need of early and targeted interventions.

### Effect of night sleep and daytime napping

For some participants, we were somewhat challenged in defining the precise time of wake-up. However, based on subjective information provided in the parental questionnaire, we found high agreement between times of wake-up based on IMI and information provided by parents. Sleep time, however, occurred later when recorded by IMI compared to parents’ reports. We hypothesize that differences could be due to parents reporting snuggle time instead of time where children actually fell asleep. We made great effort in trying to include all waking hours in the present study, and we believe that only limited time during morning hours and evening hours, which typically would be characterized by low PA levels and abundant SB time with the potential to downgrade out-of-daycare PA levels, falsely could have been eliminated from our analyses. Inspections of PA graphs for each day of monitoring (not shown) indicated that the children’s sleep was characterized by some small-scale “activity”, most likely from tossing and turning in bed. Thus, standard non-wear filters, as provided by various accelerometer software programs, may be insufficient to cut out time spent sleeping without simultaneously excluding low PA during waking hours.

No time-matched daytime napping data were available; however, we noted that on-and-off body movements and tossing and turning in the bed were observed in data files at time points where daytime napping roughly was expected to occur in subjects who reported daytime napping. This made it impossible for us to define valid times of falling asleep and wake-up when taking a nap based on the IMI approach. Therefore, higher PA levels as observed during early afternoon in children napping around noon compared to children not napping at noon most likely reflect that children either participated in high intensity playtime after lunch or were sleeping, and perhaps some of the nap time was misclassified as SB time. Short sleep duration has been found to be associated with increased risk of childhood obesity [37]. Since daytime napping is natural during preschool years [38], naps may likewise be hypothesized to be beneficial for child health. On the other hand, it is far from clear how much of daytime napping children actually spend sleeping. Thus, we believe it is a discussion point whether or not napping during the day should be excluded from final analyses to avoid misclassification as SB time. We suggest that future studies carefully consider how to handle daytime napping when analyzing PA data.

### Strengths and limitations

A major strength in the present study is that PA was assessed objectively with high wear compliance and that PA analyses encompassed all waking hours as defined by the IMI approach based on 24 h registration. Use of sophisticated time-stamped data processing based on the detailed information provided by parental logs, including institution check-ins and check-outs for each child, made it possible for us to isolate PA and SB outcomes in a number of relevant everyday settings, including time in and out of daycare.

The individual daycare has previously been identified as an important predictor of PA [35, 39, 40]. We did not take into account the specific daycare center in our analyses since the unit of recruiting in our study was the single child and not clusters of institutions. In the area from which the children were recruited there are 400–500 daycare centers. Thus, children who were enrolled in our study attended numerous different daycare institutions, which have contributed to more robust findings.

We did not distinguish between non-DC-days during weekdays and weekend days due to statistical power considerations. This could potentially have masked difference in children’s behaviors across these day types. However, weekdays for which parents reported that children did not attend daycare had more similarities with weekend days than with weekdays where parents reported that children were in daycare (data not shown). It was a limitation that it was not possible for us to discriminate between children’s time spent indoors and outdoors during daycare as time spent outdoors has been described as a predictor of preschool PA [28].

Accelerometer-determined intensity thresholds are a major issue when trying to quantify the minutes spent in specific PA intensities, and basically no uniform consensus exists regarding which cut-points are best to use in order to estimate valid amounts of time that children of different ages spend in different PA intensities during free living. However, relative differences in PA levels and SB across different everyday settings can still be meaningfully described if one recognizes the limitation of the cut-point approach.

Specific activities undertaken during time in SB and MVPA were not considered in the present study. However, supplementing accelerometer data with more detailed contextual information could provide further valuable insights into which contexts and settings children typically engage in SB and low/high PA intensities. As in any other study where PA is assessed using hip-worn accelerometers, the inability to capture cycling, swimming, and loadbearing activities correctly is a limitation to our study. We speculate, however, that generally these types of behaviors only constitute relatively small parts of preschoolers’ total habitual PA.

Finally, the children in the SKOT cohort are primarily from well-educated, high-income families. Danish preschoolers’ PA levels have previously been found not to be associated with either household income [40] or parental educational level [39]. The association between socio-economic status and PA may not emerge until school age, but we cannot rule out the possibility that generalizability of the results observed may not extend beyond the type of population sampled in the present study (i.e., majority of children from families with a high socio-economic status).

## Conclusions

Our study confirms that preschoolers’ PA and SB vary considerably during the day and that daycare represents an important setting for PA as children’s levels of PA and SB were generally more favorable in daycare than out of daycare. All children fulfilled the recommendation of 3 h of PA with at least light intensity per day, and 72% and 89% of girls and boys, respectively, accumulated at least 60 daily minutes of MPVA. Less MVPA minutes were accumulated on days with no daycare attendance compared to days with daycare attendance, especially in boys.

Our findings indicate that public health policy makers and planning of future PA initiatives in preschoolers should consider that non-DC-days and time before and after daycare attendance in particular seems to hold potential for increasing children’s PA. We consistently observed noteworthy differences in the amount of time spent physically active between the most active and least active children across all settings, and since PA behaviors are known to track into later life, targeting of PA in all contexts should optimally be favored in the least active children.

## Additional files

Additional file 1: Table S1. Subject’s characteristics, included in analyses vs. non-included. Comparisons of characteristics between subjects included in analyses and non-included subjects. (PDF 44 kb)

Additional file 2: Table S2. Wear-time (hours/day) and mean total physical activity levels across settings. Data are stratified by sex and children napping and not napping during daytime, respectively. (PDF 19 kb)

## Abbreviations

BMI: Body mass index
CPM: Counts per minute
DC-days: Daycare-days
IMI: Individual manual inspection
MVPA: Moderate-to-vigorous physical activity
Non-DC-days: Non-daycare-days
PA: Physical activity
SB: Sedentary behavior
SD: Standard deviation
