# Supplementary material for: Descriptive analysis of preschool physical activity and sedentary behaviors – a cross sectional study of 3-year-olds nested in the SKOT cohort
Source: BMC Public Health. 2017 Jun 30;17:613. doi: 10.1186/s12889-017-4521-3 (PMC5493126; doi:10.1186/s12889-017-4521-3)
Supplement: Supplementary file 1 — Subject’s characteristics, included in analyses vs. non-included. Comparisons of characteristics between subjects included in analyses and non-included subjects. (PDF 44 kb) [file 12889_2017_4521_MOESM1_ESM.pdf]

Supplementary Table 1. Subject's characteristics, included in analyses vs. non-included

	**Included**	**Non-included**	**P**
	(n=231)	(n=39)	
Age (months)	36.4±1.06	37.0±128	0.2
Sex (% boys)	49.4	54.0	0.4
Height (cm)	95.7±3.42	97.0±3.51	0.04
Weight (kg)	14.5±1.51	15.0±1.45	0.002
Body mass index (kg/m^2)	15.8±1.14	16.5±0.96	0.0006
Body mass index-for-age-Z	0.23±0.86	0.77±0.72	0.0004
% overweight (BMI$_{♂}$>17.9, BMI$_{♀}$>17.6	6.5	11.1	0.3
% obese (BMI$_{♂}$>19.6, BMI$_{♀}$>19.4	0	0	
Highest educational level, mother			
Gymnasium/high school or lower secondary school education (%)	3.9	9.1	0.10
Trainee or vocational education (%)	8.7	14.1	
Short academic education (<3 years) (%)	11.3	6.1	
Medium length academic education (3-4 years) (%)	35.5	30.3	
Long academic education (>4 years) (%)	40.7	40.4	

Data are means ±standard deviations unless stated otherwise. Differences between included vs. non-included subjects are tested by un-paired t-tests or Pearson's Chi-squared-test as appropriate.
