# Supplementary material for: Descriptive analysis of preschool physical activity and sedentary behaviors – a cross sectional study of 3-year-olds nested in the SKOT cohort
Source: BMC Public Health. 2017 Jun 30;17:613. doi: 10.1186/s12889-017-4521-3 (PMC5493126; doi:10.1186/s12889-017-4521-3)
Supplement: Supplementary file 2 — Wear-time (hours/day) and mean total physical activity levels across settings. Data are stratified by sex and children napping and not napping during daytime, respectively. (PDF 19 kb) [file 12889_2017_4521_MOESM2_ESM.pdf]

Supplementary Table 2. Wear-time (hours per day) and mean total physical activity levels across settings

			Wear time		Mean total PA (CPM)	
	n	n_{nap}	nap-	nap+	nap-	nap+
**Girls**						
All days, overall	113	73	12.4±0.9	12.6±0.7	562±138	506±121*
Sick days	11	8	12.5±2.3	12.2±2.3	260±17	340±138
DC-day	92	59	12.5±0.8	12.6±1.1	583±139	518±146*
DC-day, before DC	92	59	1.6±0.7	1.6±0.6	337±120	359±115
DC-day, in DC	93	59	6.7±1.0	6.3±1.3	636±198	544±195*
DC-day, after DC	92	59	4.2±1.1	4.7±1.2**	569±163	520±172
Non-DC-day	109	72	12.4±1.3	12.6±0.7	560±165	499±133**
**Boys**						
All days, overall	109	81	12.6±0.8	12.6±0.8	569±83	587±136
Sick days	10	8	12.8±1.1	12.1±1.1	492±87	429±199
DC-day	92	68	12.6±1.0	12.5±1.0	596±102	594±143
DC-day, before	91	67	1.6±0.6	1.7±0.6	398±146	368±112
DC-day, in DC	92	68	6.8±1.2	6.4±1.2	692±137	640±189
DC-day, after DC	92	68	4.2±1.0	4.5±1.0	508±107	598±250*
Non-DC-day	107	79	12.5±1.1	12.6±1.1	540±109	571±159

CPM: Counts per minute, DC: Daycare, n_{nap}: indicates number of children napping during daytime, nap-: children not napping during daytime, nap+: children napping during daytime. *: significant different compared with children not napping during daytime.
**: borderline significant different compared with children not napping during daytime.
